# Even a single positive blood culture may matter – A case of prosthetic valve infective endocarditis caused by *Corynebacterium kroppenstedtii*

**DOI:** 10.1016/j.idcr.2024.e02049

**Published:** 2024-07-31

**Authors:** Adam Cewers, Torgny Sunnerhagen, Patrik Gilje, Fredrik Wannheden, Jonas Bläckberg, Per Wierup, Mårten Larsson, Magnus Rasmussen

**Affiliations:** a Division of Infection Medicine, Helsingborg Hospital, Helsingborg, Sweden; b Division of Infection Medicine, Department of Clinical Sciences Lund, Lund University, Lund, Sweden; c Department of Clinical Microbiology, Infection Control and Prevention, Office for Medical Services, Region Skåne, Lund, Sweden; d Department of Clinical Microbiology, Copenhagen University Hospital, Rigshospitalet, Copenhagen, Denmark; e Department of Cardiology, Clinical Sciences, Lund University and Skane University Hospital, SE-221 85 Lund, Sweden; f Department of Clinical Physiology, Clinical Sciences, Lund University, SE-221 85 Lund, Sweden; g Department of Infectious Diseases, Skåne University Hospital, Lund, Sweden; h Department of Cardiothoracic and Vascular Surgery, Skåne University Hospital, Lund, Sweden

**Keywords:** Infective endocarditis, Valve culture, Surgery

## Abstract

*Corynebacterium* is a skin commensal bacterium that can contaminate blood cultures. It is however also a rare cause of infective endocarditis (IE). Here we report a case of *Corynebacterium kroppenstedtii* aortic prosthesis IE in a 76-year-old man where only a single blood culture bottle was positive initially. *C. kroppenstedtii* is a very rare cause of IE, only reported two times previously. The diagnosis in our case was confirmed by repeated blood culture positivity and eventually by detection of DNA from *C. kroppenstedtii* on heart valves after valve exchange surgery. At surgery an aortic root abscess was detected and the valve was replaced by a homograft. Recovery was complicated by antibiotic-induced nephrotoxicity and treatment was concluded with moxifloxacin in combination with rifampicin. Recovery was uneventful. This case demonstrates that growth in even a single blood culture bottle may be important in patients with prosthetic heart valves.

## Introduction

*Corynebacterium* is a genus of Gram-positive rods with multiple species, belonging to natural skin microbiota. Finding of these bacteria in blood cultures are therefore often considered as nonpathogenic contamination. However, there are studies indicating that bacteremia with *Corynebacterium* represent true infection in as much as 44–71 % of cases [1], [2], [3], [4], [5]. In infective endocarditis (IE) caused by *Corynebacterium,* studies suggest that two species are more frequently involved, namely *Corynebacterium jeikeium* and *Corynebacterium striatum. Corynebacterium* species typically cause IE in patients with prosthetic heart valves [4], [6], [7].

*Corynebacterium kroppenstedtii* is a rarely encountered species in human infections. In one study of 330 episodes of *Corynebacterium* bacteremia, *C. kroppenstedtii* was only found in one episode and the bacterium was deemed to be a contamination in that case. As opposed to other pathogens within this genus, *C. kroppenstedtii* lacks mycolic acid in the cell wall [8]. This species has previously been reported to cause mastitis [9]. To our knowledge, IE caused by *C. kroppenstedtii* has only been described in two patients previously, one with a native valve and one with an aortic valve prosthesis [10], [11].

*Corynebacterium* is typically sensitive to vancomycin which is often used for treatment of IE caused by this genus. The bacteria are sometimes also sensitive to beta lactam antibiotics which is the preferred treatment in such cases. Moreover, corynebacteria are often sensitive to aminoglycosides and rifampicin which are often added to the treatment [6].

In this report, we describe a case of prosthetic valve IE caused by *C. kroppenstedtii* where, initially, only a single blood culture bottle was positive. The diagnosis was confirmed upon repeated blood cultures and surgery with finding of DNA from *C. kroppenstedtii* on the heart valve*.*

## Case presentation

**Fig. 1:**
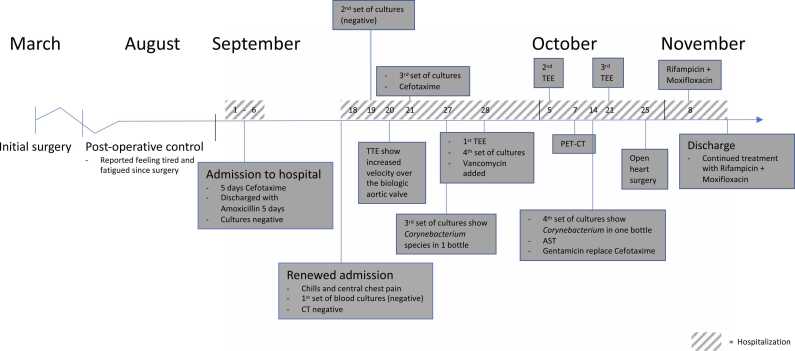
Time-line describing the evolution of this case. Abbreviations used were; TTE, transthoracic echocardiography; TEE, transesophageal echocardiography; PET-CT, positron emission tomography–computed tomography; AST, antibiotic susceptibility testing.

A 76-year-old man presented to a local hospital in our region in September 2022 with fever of 40 °C and chills. The patient had been operated in March 2022 with insertion of a biologic aortic valve prosthesis due to aortic stenosis. In addition to the valve disease, the patient had atrial fibrillation, hypertension, spinal stenosis, polymyalgia rheumatica, a history of kidney stones and stroke without sequelae. He was on treatment with apixaban and 5 mg of prednisolone daily. On post-operative control in August, the patient had disclosed that he felt tired. Upon clinical examination in September, no focal infection was identified and blood cultures were negative. Treatment with cefotaxime for five days intravenously followed by five days of amoxicillin 750 mg tid was given and the patient recovered. A time-line describing the case is shown in Fig. 1.

Five days after finishing the amoxicillin course, the patient returned to the hospital with complaints of fever, nocturnal chills, central chest pain and weakness in both arms. Pulse was 100 beats per minute, blood pressure was 155/90 mmHg while oxygen saturation and respiratory rate were within the normal ranges. A systolic murmur was present. C-reactive protein (CRP) was 105 mg/L, B-white blood cell count (WBC) was 13.8 × 10^9^/L, P-Troponin I (TnI) 423–488-260 ng/L.

Antibiotics were not given immediately since the patient was deemed stable. Computerized tomography (CT) with intravenous contrast of the thorax was normal. Blood cultures obtained the first day of admission were negative and repeated cultures the following day were also negative. Transthoracic echocardiogram (TTE) two days after admission showed no signs of vegetation on the heart valves, but increased velocity over the biologic aortic valve was observed.

**Fig. 2:**
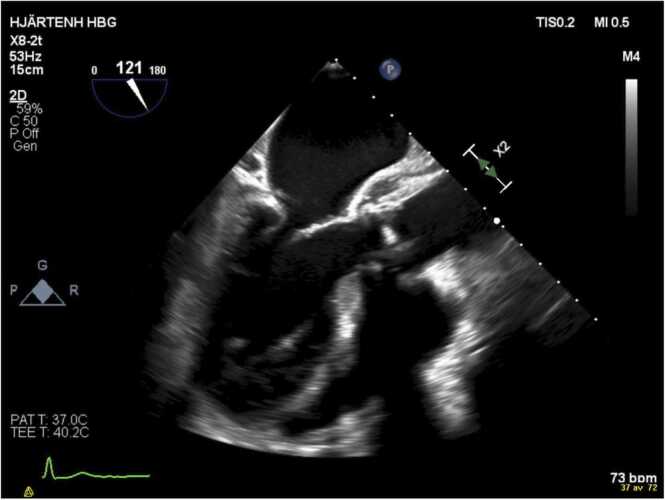
Transesophageal echocardiography of the patient on day 10 of hospitalization.

Due to continued fever and rising CRP (maximum 263 mg/L), cefotaxime at two grams tid was instituted after renewed blood cultures (the third set). Nine days after admission *Corynebacterium* grew in one blood culture bottle from a third set of blood cultures obtained three days after admission. This was first deemed as a possible contamination. Transesophageal echocardiography (TEE) was performed on day 10 of the hospitalization demonstrating thickening of the aortic valves compatible with vegetations. Moreover, TEE visualized changes in the aortic root suggestive of abscess (Fig. 2). New blood cultures were secured and again a *Corynebacterium* was found in 1 out of 4 bottles. Identification was performed with MALDI-TOF MS (Bruker Daltonics) with a top score of 1.8 for *C. kroppenstedtii* (using the Bruker MBT Compass software and MBT DB10833 library)*.* At this point vancomycin was added to the treatment aiming at trough values of 15–20 mg/L. Antibiotic susceptibility testing was done and interpreted according to EUCAST recommendations and breakpoints using disk diffusion for benzylpenicillin, linezolid, clindamycin, tetracycline, moxifloxacin, and rifampicin, and using Etests (bioMérieux) according to the manufacturer’s instructions for vancomycin, gentamicin, daptomycin and cefotaxime. The *C. kroppenstedtii* isolate tested sensitive for all antibiotics where EUCAST give species specific breakpoints, except for benzylpenicillin for which the isolate tested resistant. For gentamicin, daptomycin and cefotaxime, where EUCAST breakpoints do not exist, the MIC values were 0.032 mg/L, 0.5 mg/L and 8 mg/L respectively. Upon receipt of the AST, the treatment was changed to vancomycin and gentamicin (3 mg/kg once daily).

**Fig. 3:**
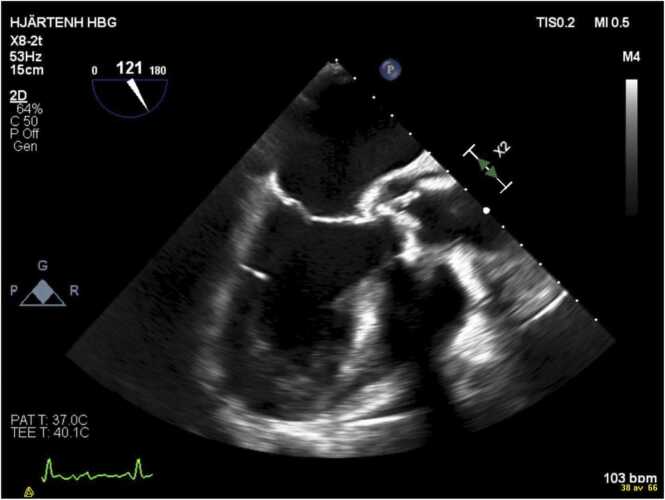
Transesophageal echocardiography of the patient on day 18 of hospitalization.

**Fig. 4:**
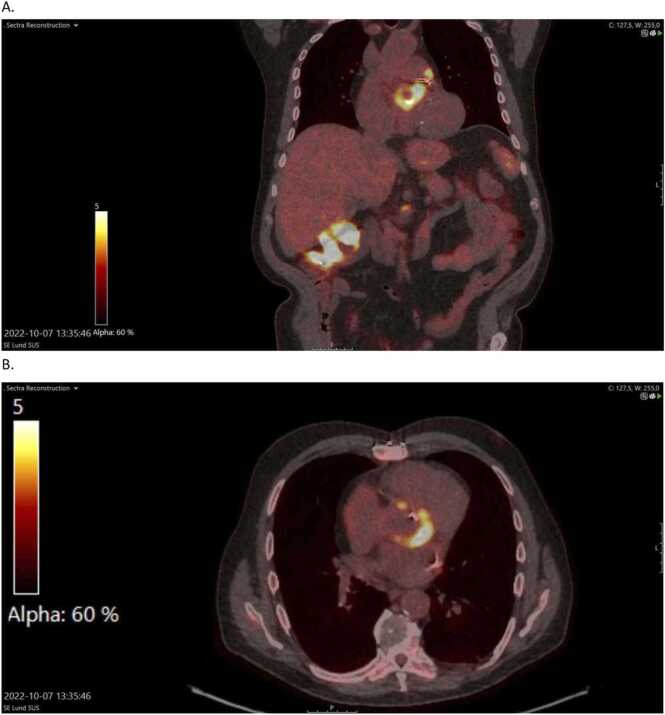
^18^F-fluorodesoxyglucose positron emission tomography–computed tomography (FDG PET-CT) at day 20 of hospitalization. A shows a coronary view and B a transversal view.

The endocarditis team at Skåne University hospital was consulted and suggested further work-up with ^18^F-fluorodesoxyglucose positron emission tomography–computed tomography (PET-CT) and renewed echocardiography due to an uncertainty if the changes in the aortic root represented abscess or post-operative changes. A new TEE on day 18 of admission was performed showing progression in both the thickening of the leaflets as well as in the size of the aortic root cavity, compatible with IE and abscess. As in the previous TEE, no mobile vegetations were observed (Fig. 3). PET-CT was performed 19 days after admission and was consistent with IE (Fig. 4). CRP-values were still, day 20 of treatment, around 150 mg/L. A third TEE was performed 33 days after admission that showed further progression of the suspected abscess of the aortic root. At this time, the patient developed kidney failure and trough vancomycin concentrations rose to 31 mg/L. Gentamicin was discontinued and treatment with vancomycin at adjusted dosage was continued. The patient underwent open heart surgery on day 37 of hospitalization with complete removal of the infected biological aortic valve and thorough debridement and exteriorization of the peri-annular abscess extending from underneath the non-coronary sinus through the left coronary sinus. The aortic root was reconstructed using homograft as replacement with coronary artery reimplantation. Perioperative inspection revealed an abundance of vegetations on the biologic valve and abscess of the left and non-coronary cusp. 16S rRNA gene PCR and sequencing revealed the presence of DNA from *C. kroppenstedtii* with a 100 % identity match using BLAST (543/543 base pairs)*.* Cultures from the valves were negative. Postoperatively an atrio-ventricular block grade III developed necessitating the insertion of a pacemaker. At time of planned discharge, two weeks post-operatively, the patient again developed kidney failure why vancomycin was discontinued and the patient instead received per oral treatment with rifampicin 600 mg in combination with moxifloxacin 400 mg once daily for an additional four weeks. In total, 69 days of effective antibiotics was administered. The treatment of cefotaxime is not included in this calculation as the high MIC of the isolate likely made treatment with this antibiotic ineffective.

During hospitalization the patient had suffered prolonged uncontrolled infection leading to weight loss of approximately 10 kg, falls and one period of severe confusion. The recovery was uneventful, and the patient has not shown signs of relapse one year after conclusion of treatment.

## Discussion

This case demonstrates that the finding of a bacterium even in a single blood culture bottle may be of high clinical significance in patients with prosthetic heart valves. The perioperative finding of an abscess and vegetations and the finding of DNA from *C. kroppenstedtii* in the valve tissue provided a secure diagnosis of definite IE in this case, confirming that the single positive blood culture bottle represented true bacteremia caused by IE [12]. *C. kroppenstedtii,* appears to be a very rare cause of IE and our case is only the second case of prosthetic valve IE described with this bacterium. Therefore, from a bacteriological point of view it was understandable that the finding was first suggested to be a skin contamination.

This case also shows that repeated blood cultures may be of value to diagnose severe infections like IE. Despite several days of intravenous antibiotics, though with cefotaxime which the isolate had high MIC values to, another blood culture bottle grew *C. kroppenstedtii* again. Thus, the major microbiology criterion of the modified Duke criteria was fulfilled [13].

In this case, cefotaxime was administered before identification of the pathogen and continued also after *C. kroppenstedtii* was identified since the finding was first was considered a contamination. After identification of the pathogen, vancomycin was administered intravenously in accordance with available data regarding treatment of IE caused by *Corynebacterium* species [6]. When the AST was available, treatment continued with vancomycin and gentamicin, in accordance with Swedish guidelines. The patient developed kidney failure, most likely due to the aminoglycoside why this treatment was discontinued. Daptomycin was a theoretical option for treatment with the isolate displaying low MIC. However, daptomycin is considered problematic to use for *Corynebacterium* IE since there are several reports about development of resistance during treatment [14], [15]. Instead, a combination therapy of rifampicin and moxifloxacin was chosen which also enabled outpatient treatment.

Contributing factors for the delayed diagnosis of IE in this patient included the apparent low virulence of the pathogen. Most likely, the bacterium was introduced during the patients’ aortic valve surgery, six months before admission. The fact the patient had felt tired since this operation is compatible with an early infection. The clinical symptoms were however concordant with a more acute onset of infection with high fever, chills and central chest pain. Therefore, we cannot exclude that the infection was caused by hematogenous spreading of the bacteria to the aortic valve. Another factor affecting the delay between diagnosis of IE and surgery were the ambiguities of the imaging. Some changes were initially believed to represent postoperative changes but upon surgery, the IE diagnosis was confirmed both macroscopically and microbiologically. Vegetations or new onset of paravalvular leak are highly suspicious of IE but the patient herein did not display such changes. Our patient initially instead presented with more subtle findings that were hard to distinguish from normal postoperative findings. For example, a localized swelling of the annular structures frequently represents annular infection, but if the area has been packed with procoagulant material at time of surgery, this will potentially mimic an early infection. However, the only area of the annulus where procoagulant material can be applied is the non-coronary sinus, eg between the aortic and mitral valve. Moreover, a generalized thickening of the leaflet tissue, as was seen in this case, is indicative of IE but is also seen with early valve thrombosis, which benefits from anticoagulant treatment. By reviewing the imaging findings over time and through repeated discussions in the endocarditis team we came to the decision to subject the patient to valve replacement surgery. Given the perioperative findings this was a correct measure.

We conclude that the single blood culture with *C. kroppenstedtii* was the clue that eventually lead to the successful treatment of this patient. A positive blood culture in patients with a heart valve prosthesis should always be carefully considered even if the bacterium is potential contaminant.

## Ethical approval

Not applicable to case reports in Sweden.

## Funding

None.

## Consent

The patient gave his informed to consent to the writing of this report.

## CRediT authorship contribution statement

**Fredrik Wannheden:** Methodology, Writing – review & editing. **Mårten Larsson:** Methodology, Writing – review & editing. **Per Wierup:** Methodology, Writing – review & editing. **Jonas Bläckberg:** Methodology, Validation, Writing – review & editing. **Patrik Gilje:** Methodology, Visualization, Writing – review & editing. **Torgny Sunnerhagen:** Methodology, Writing – review & editing. **Adam Cewers:** Data curation, Investigation, Visualization, Writing – original draft. **Magnus Rasmussen:** Conceptualization, Data curation, Formal analysis, Investigation, Methodology, Project administration, Resources, Software, Supervision, Writing – original draft, Writing – review & editing.

## Declaration of Competing Interest

None.
